# The Blow-Off Impulse Equivalence of Typical Missile Homogeneous Al-Alloy under Multienergy Composite Spectrum Electron Beam and Powerful Pulsed X-ray

**DOI:** 10.3390/ma14175002

**Published:** 2021-09-01

**Authors:** Dengwang Wang, Yong Gao, Wei Chen, Shanghui Yang, Jing Zhang, Jie Wang, Sheng Wang

**Affiliations:** 1 Department of Nuclear Science and Technology, Xi’an Jiaotong University, No.28, Xianning West Road, Xi’an 710049, China; wdw21s@stu.xjtu.edu.cn (D.W.); gaoyong1108@stu.xjtu.edu.cn (Y.G.); y574055234@stu.xjtu.edu.cn (S.Y.); zhangjing1108@stu.xjtu.edu.cn (J.Z.); wangjie1@xjtu.edu.cn (J.W.); 2 Northwest Institutes of Nuclear Technology, No.28, Pingyu Road, Xi’an 710024, China; chewei@nint.ac.cn

**Keywords:** homogeneous Al-alloy, blow-off impulse, electron beam, powerful X-ray, equivalence

## Abstract

The electron beam, one of the most effective approaches to simulate the irradiation effects of powerful pulsed X-ray in the laboratory, plays an important role in simulating the thermodynamic effects of powerful pulsed X-ray. This paper studies the thermodynamics equivalence between multienergy composite spectrum electron beam and blackbody spectrum X-ray, which is helpful to quickly determine the experimental parameters in the simulation experiment. The experimental data of electron beam are extrapolated by numerical calculation, to increase the range of energy flux. Through calculating the blow-off impulse of blackbody spectrum X-ray irradiation, we obtained the curve of X-ray blow-off impulse varying with energy flux, and then found two categories of equivalent relations—equal-energy flux and equal-impulse—by analyzing the calculation results of electron beam and X-ray blow-off impulse. Based on such relations, we could directly or indirectly obtain the results of blackbody spectrum X-ray irradiation blow-off impulse via electron beam experiment.

## 1. Introduction

Powerful pulsed X-ray is mainly soft X-ray, featuring high-energy flux and short duration (about 100 ns) [1,2]. Under the irradiation of powerful pulsed X-ray, the materials within the optical thickness on the side under radiation will melt rapidly, vaporize, or even partially dissociate into plasma, and blow off against the light at a high speed. The generated blow-off impulse can cause the buckling deformation and vibration of the structure, leading to its instability [3]. At the same time, due to the inhomogeneous X-ray energy deposition, inside the material will emerge thermal shock waves whose propagation and reflection can cause cylindrical shell damage and spallation damage [4]. These problems caused by powerful pulsed X-ray irradiation are collectively referred to as thermodynamic effects. Therefore, studying the thermodynamic effects of powerful pulsed X-ray is of great significance to assess the survivability of spacecraft and test the effectiveness of antinuclear reinforcement measures [5].

The thermodynamic effects of material and structure can be induced by powerful pulsed X-ray [6], electron beams [7], laser beams [8], and chemical explosion [9,10]. However, although the wavelength of the high-energy laser is usually in the infrared band and much longer than the X-ray, its optical thickness is usually close to zero for opaque medium. Therefore, laser is usually treated as a heat flow boundary condition in the study of laser-induced hard damage. The thermodynamic effects of powerful pulsed X-rays are studied indirectly by means of a low-energy, intense-current, pulsed electron accelerator [11,12]. Compared with powerful pulsed X-ray, the optical thickness of electron beam is relatively large, and the peak and gradient of energy deposition profile are relatively small. Therefore, it is necessary to further study the equivalence of electron beams and powerful pulsed X-ray from the aspects of the energy spectrum and irradiation mode. Yang [13] studied the energy deposition profile of three monoenergetic electron beams in aluminum alloy materials at different angles of incidence and found that the irradiation effects of powerful pulsed X-rays could be well simulated when the low-energy electron beams had a larger angle of incidence on the target material.

Relativistic Electron Beam (REB) is a new technology developed to meet the needs of nuclear fusion. It has been applied in high-energy-density physics, nuclear radiation simulation, and material science since the 1980s. The United States and the former Soviet Union [14,15] attach great importance to the research of high-power pulsed electron beam technology. For example, the United States has successively built several pulsed electron beam accelerators with higher power levels. Intense pulsed light ion beams with pulse width of 10–1000 ns, current intensity of 10–1000 kA, and particle energy of 10 KeV–10 MeV have been produced in the strong pinch electron beam diode. Studies on the effects of irradiating materials with electron beams have been carried out since the 1980s and applied in practice since the 1990s.

To sum up, electron beam is an effective method to simulate powerful pulsed X-ray [16,17,18,19,20]. However, it is necessary to study the equivalence of the above two for the thermodynamic effects caused by the radiation mechanism difference between them. When the energy flux of X-ray radiation is high enough, the vaporization and melting of the material on the light front and the outward ejection of high-temperature and high-pressure material would exert a recoil impulse (blow-off impulse) on the structure. On the one hand, the blow-off impulse exerts a compression wave (which belongs to the material response) to the material (in this case, the exposed surface will not produce sparse tensile wave due to the suppression of the light-facing surface). On the other hand, the blow-off impulse may vibrate spatial structures, thus leading to permanent deformation and even buckling. Therefore, the blow-off impulse is one of the key parameters of X-ray radiation thermodynamics. In this paper, the study on the equivalence of electron beam and X-ray, or the study on the equivalent relationship between multienergy composite spectrum electron beam and blackbody spectrum X-ray in terms of thermodynamic effects, could provide a reference for the rapid determination of experimental parameters for simulation experiment.

## 2. Experimental System and Numerical Method

### 2.1. Energy Spectrum of Powerful Pulsed X-ray

Powerful pulsed X-ray is radiated outward as an expanding X-ray fireball, which can be approximated as a blackbody [21,22,23]. Therefore, the X-ray energy spectrum is approximated as the blackbody radiation spectrum. Blackbody spectrum X-ray refers to the energy radiated outward in the form of electromagnetic waves by high-temperature objects. The X-ray energy and the proportion of photon component vary with the temperature of the objects. The radiation intensity is computed by Planck formula.

(1) Bν=2πhν3c21ehνkT−1

**Figure 1:**
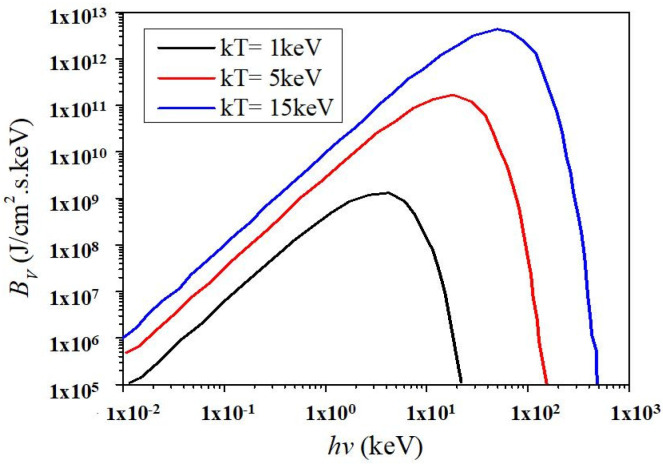
Blackbody spectrum radiation intensity at different temperatures.

*h* denotes Planck constant (*h* = 6.62 × 10^−34^ J·s), *ν* is photon frequency, *c* is light speed, *k* is Boltzmann constant (*k* = 1.38 × 10^−13^ J/K), *T* represents blackbody temperature, *hν* is photon energy (keV), and *kT* is blackbody temperature (keV). Figure 1 shows the blackbody spectrum radiation intensity at different temperatures.

The normalized spectrum is usually employed practically, and the formula is written as

(2) bν=BνσT4=15h4ν3π4k4T41ehνkT−1

### 2.2. Platform for Electron Beam Experiment

The pulsed electron accelerator is used to simulate the X-ray’s thermodynamic effects. The analysis of the radiation loading characteristics of the Relativistic Electron Beam (REB) is shown as below.

**Figure 2:**
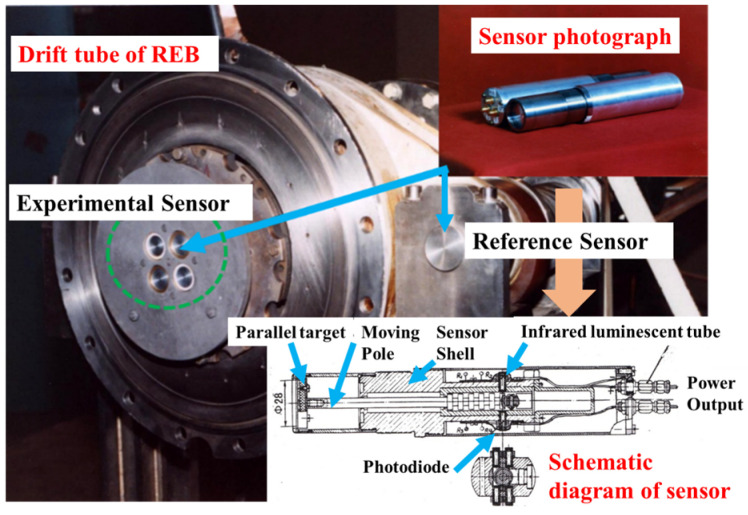
Impulse sensors installed in the drift tube of REB.

REB is a low-impedance relativistic electron beam accelerator used for nuclear weapons effect simulations (Figure 2). The accelerator system consists of a Marx generator, a water–dielectric coaxial line, a diode, and further auxiliary equipment. The test running at DC charge voltage is ±55 kV and ±70 kV, and the maximum output current is 600 kA with a tube voltage of 1.2 MV. The energy (*E*_0_) and the average energy (*E_av_*) are 0.1~1.2 MeV and 0.5~0.6 MeV, respectively, with the Full-Width at Half-Maximum (FWHM) of 50~80 ns. When electron energy flux is less than 400 J/cm^2^, the beam spot diameter is 90~170 mm on the target. By adjusting the distance between the target and the electron beam cathode, the energy flux can reach 400~1000 J/cm^2^, and the beam spot diameter is 50~60 mm. A new method of electron beam measurement based on a miniature Faraday tube array with attenuation slices of different thicknesses is proposed to obtain the energy distribution of high-current electron beam with time domain characteristics and location distribution. The total beam intensity is measured by Rogowski coil, the current intensity is measured by Faraday array, and differential loop is used to measure REB diode voltage in the experiment.

### 2.3. Blow-Off Impulse Measurement Device

Figure 2 shows the photo and schematic structure of the blow-off impulse probe. The parallel target is attached to one end of the signaling rod whose other end is shaped into several evenly spaced rings. Moreover, infrared luminous diodes and photoelectric triodes are installed on both sides of the rod. When the blow-off impulse produced by electron radiation pushes the target and the transmitting bar, the rings block off the light and cut off the signal; after that, the signal is restored. The digital oscilloscope records the time interval “Δ*t*” while the rod moves through the spacing *L* between two rings (*L* = 5 mm). Thus, the average velocity of the rod is obtained:

(3) v=L/[Δt(1−μ)]

where *μ* is the friction adjustment factor (4.5%), which is calibrated by a gas gun. According to the law of momentum conservation, the blow-off impulse *I* can be expressed by

(4) $$f=(m_{0}-\Delta m)v$$

Then, the blow-off impulse is the momentum of the removed part normalized to the target area.

(5) $$I=(m_{0}-\Delta m)v/A$$

where *f* is momentum, Δ*m* is the mass loss of the target after radiation, *m*_0_ is the initial total mass of the target and the rod, *A* is the irradiated area (*A* ≈ 2.01 cm^2^), and *I* is blow-off impulse. The uncertain impulse is less than 10%. If the energy flux *E_Φ_* of soft X-ray radiated on the target is given, the coupling coefficient of blow-off impulse will be determined:

(6) $$\beta =I/E_{\Phi }$$

### 2.4. Thermal Shock Measurement

**Figure 3:**
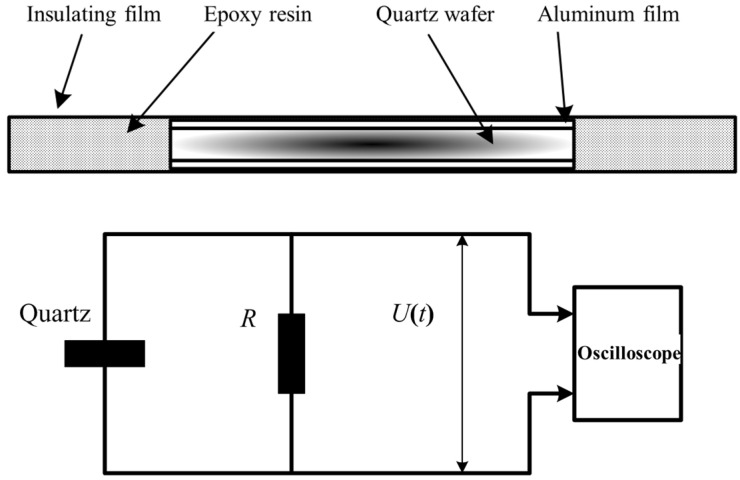
Quartz sensor and measuring circuit.

**Figure 4:**
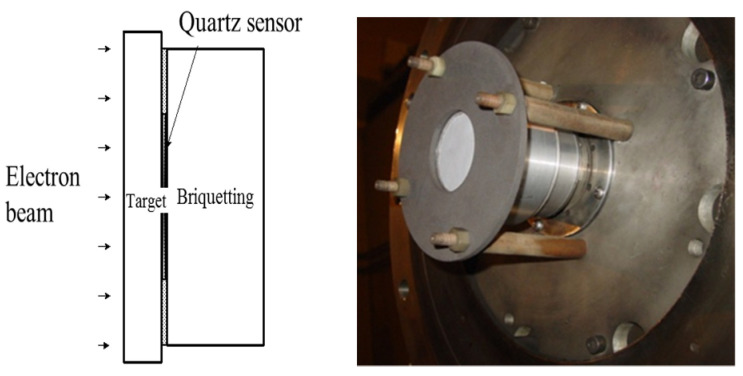
The schematic diagram and installation drawing of thermal shock sensor on REB.

For thermal shock measurement on the REB, an X-cut shunt protection ring quartz piezoelectric crystal sensor and measuring circuit (Figure 3) is used. The thermal shock sensor is installed in the same position as the impulse sensor, both installed in the REB drift tube. The sensor’s outer diameter is Φ 15 mm, the diameter of the inner electrode surface is Φ 7.5 mm and the thickness is 3 mm, the external load resistance of the sensor is 51 Ω, and the characteristic impedance of the external coaxial cable is 50 Ω. Figure 4 shows the schematic diagram and installation drawing of thermal shock sensor on REB. The target is a typical missile shell material of homogeneous Al-alloy.

When the thermal shock pressure *σ*(*t*) acts on the front electrode of the quartz sheet, piezoelectric current *I*(*t*) is generated in the measuring circuit due to the piezoelectric effect, and the piezoelectric charge *Q* is as follows:

(7) $$Q(t)=\int_{0}^{t}i(t)dt=\int_{0}^{t}U(t)/Rdt$$

where *U*(*t*) is the voltage signal on the load resistor *R*. The piezoelectric equation of quartz crystal under one-dimensional plane strain state is as follows:

(8) Q=AKσ

where *A* is the electrode area and *K* is the wafer piezoelectric coefficient. It is as follows:

(9) $$\sigma (t)=\frac{1}{AKR}\int_{0}^{t}U(t)dt$$

The above equation shows that the thermal shock wave waveform passing through the crystal can be obtained by measuring the piezoelectric voltage signal and integrating the signal.

### 2.5. Finite Element Calculation Software

Due to the failure of the existing finite element analysis software to deal with the thermodynamic problems caused by electron beam and pulsed X-ray irradiation, we used the finite element method and the FORTRAN language to compile the program RAMA [2,24] for the thermodynamic effects of pulsed beam irradiation to carry out relevant numerical simulation work. Embedding a variety of constitutive models and equations of state including the orthotropic dynamic elastoplastic constitutive model, the RAMA program can not only deal with the fracture and vaporization of materials, but simulate the stress wave propagation in the collision of two-dimensional flat plates by anisotropic and isotropic materials, and the thermodynamic effects of two-dimensional X-ray in various shapes. Thus, it has a certain practicality in engineering applications [25].

The calculation process of electron beam energy deposition can be summarized as follows:

In a unit mass thickness material *S*(*E*), the electron energy loss, mainly caused by electron collision ionization for lower-energy electrons, is as follows [26]:

(10) $$S(E)=\frac{2\pi e^{2}N_{0}Z}{m_{0}c^{2}\beta^{2}A}\left\{In[\frac{m_{0}c^{2}\beta E}{2J^{2}(1-\beta^{2})}]-[2{\left(1-\beta^{2}\right)}^{1/2}-1+\beta^{2}]\mathrm{In}2+(1-\beta^{2})+\frac{1}{8}{\left[1-{\left(1-\beta^{2}\right)}^{1/2}\right]}^{2}-\delta_{0}\right\}$$

where *N*_0_ is the Avogadro constant, *e* is the electron charge, *Z* is the atomic number, *A* is the atomic weight, $m_{0}c^{2}=0.511\;\mathrm{MeV}$ is the electron rest mass, $\beta =\left\{1-{\left[m_{0}c^{2}/(E+m_{0}c^{2})\right]}^{2}\right\}$, *E* is the kinetic energy of electron, *J* (MeV) is the average ionization energy, $\delta_{0}$ is the density effect correction factor, and $\delta_{0}$ is introduced to reduce energy loss by electron collisions through target polarization.

The next step in electronic energy is decided by the law of energy logarithmic delay.

(11) $$E_{n+1}=KE_{n}$$

*K* is 0.9576 [27].

The continuous slow-down used to approximately calculate the interval mass range of electron.

(12) $$\Delta S_{n+1}=\int_{E_{n+1}}^{E_{n}}{\left|\frac{dE}{dS}\right|}^{-1}dE$$

The electronic coordinates of step *n* + 1 are as follows:

(13) $$X_{n+1}=X_{n}+\Delta S_{n+1}\frac{\cos\theta_{n+1}}{\rho (j)}$$

where ρ(j) is the Lagrange interval density of *j*, $\theta_{n+1}$ is the positive angle between the electron motion direction, and the *x*-axis of step *n* + 1.

According to the Moliere Multiple Scattering Theory, the scattering angle is as follows [28]:

(14) $$F(\theta )=f^{\left(0\right)}(\theta )+f^{\left(1\right)}(\theta )/B+f^{\left(2\right)}(\theta )/B^{2},\omega =X_{c}B^{1/2}\theta$$

where *B*, *Xc* is one of energy parameters, and θ is the discrete angle.

The electronic polar angle is as follows:

(15) $$\theta_{n+1}=\cos^{-1}(\cos\theta_{n}\cos\omega +\sin\theta_{n}\sin\omega \cos\varphi )$$

where φ is the electronic azimuth and ω is the determined uniform sampling.

If the deposition energy of each incident electron is $\Delta E_{i}$ in the interval *J*, the total deposition energy of each incident electron is as follows:

(16) $$E_{n}=\sum_{i=1}^{N}\Delta E_{i}$$

The specific energy of unit energy flux in interval *J* is as follows:

(17) $$Q_{J}=\frac{E_{n}}{\rho_{0}\Delta xN}$$

where Δx is the mesh step.

According to the current and voltage waveform of experiment, the energy deposition of electron beam can be calculated, as shown below:

(18) $$E_{R}(x,t)=U_{t}I_{t}Q_{J}\Delta T/S$$

where $U_{t}$, $I_{t}$ are the instantaneous voltage and current of REB diode; ΔT is the time step; and *S* is the spot area of electron beam on the target.

## 3. Error Analysis of Numerical and Measurement

### 3.1. Analysis of Mesh Independence

The numerical results are determined by grid sizes [28]. A grid-independent has almost unchanged numerical results, i.e., the encrypted grid slightly affects the numerical results. Numerical calculation should be grid-independent, which is also essentially required for the international academic community to accept numerical calculation [29,30]. Grid-independent results have associations with specific physical problems, which should be determined by trial calculation according to specific problems. Overall, the grid can be calculated from large to small and is capable of comprehensively considering the precision and speed of the calculated results. Thus, the appropriate mesh size is critical for numerical calculation.

X-ray energy decays exponentially in the material when irradiating the target. X-ray energy is primarily deposited as a thin layer on the surface of a material [31]. Besides, the X-ray energy deposition profile varies rapidly with the depth of the thin layer. Accordingly, different mesh sizes significantly impact the distribution of the X-ray energy deposition, which critically determines blow-off impulse calculation. The numerical results of blow-off impulse are largely determined by grid size, so the irrelevant grid should be developed, and the grid-independent solution should be obtained.

**Figure 5:**
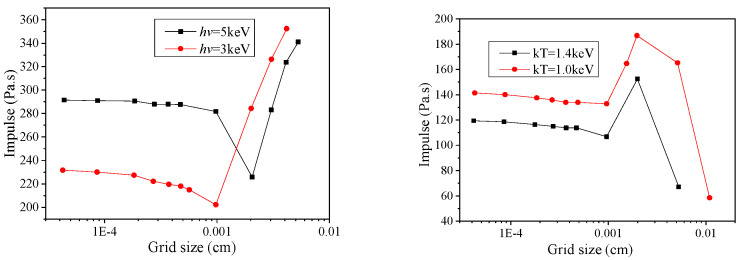
Effect of grid size on blow-off impulse.

Figure 5 illustrates the result of blow-off impulse when monoenergetic and blackbody X-rays irradiate aluminum target by different mesh sizes. The effect of mesh size on impulse results is obviously shown. Blow-off impulse varies with the melting or gasification of a material, which was not discussed in this study. The mesh size was 0.0005 cm to impulse and computational efficiency, and the appropriate time step was adopted given the finite difference stability condition.

### 3.2. Measurement Errors

The calculation formula of impulse sensor is as follows [32,33]:

(19) $$I=m_{0}L/\left[(1-\Delta t_{p}/\Delta t_{m})\Delta t_{m}A]=I(m_{0},L,\Delta t_{m},A\right)$$

According to the function propagation theory of measurement uncertainty of indirect measurement, the relative measurement uncertainty of measured eruption impulse is as follows:

(20) $$\delta_{I_{1}}\leq \pm {\left\{\delta_{m_{0}}^{2}+\delta_{L}^{2}+\delta_{A}^{2}+{\left[\delta_{\Delta t_{m}}/\left(1-\Delta t_{p}/\Delta t_{m}\right)\right]}^{2}\right\}}^{1/2}$$

where, $\delta_{m_{0}}$, $\delta_{L}$, $\delta_{\Delta t_{m}}$, and $\delta_{A}$ are the relative measurement uncertainties of direct measurement quantities *m*_0_, *L*, Δ*t_m_*, and *A.*

When *m*_0_ = 30.5 g, *L* = 0.6 cm, Δ*t_m_* = 0.5 ms, *A* = π⋅192 cm^2^, and $\Delta t_{p}/\Delta t_{m}=4.5\%$. According to the actual situation of the field measurement system, the relative uncertainty of each direct measurement quantity can be estimated as

(21) $$\delta_{m_{0}}\leq \pm \frac{\left|\Delta m\right|}{m_{0}}=\pm \frac{0.03}{30.5}=\pm 0.1\%;\;\delta_{L}\leq \pm \frac{\left|\Delta L\right|}{L}=\pm \frac{0.025}{0.6}=\pm 4.2\%$$

(22) $$\delta_{\Delta t_{m}}\leq \pm \frac{\left|\Delta \left(\Delta t_{m}\right)\right|}{\Delta t_{m}}=\pm \frac{0.06}{0.5}=\pm 12\%;\;\delta_{A}\leq \pm \frac{\left|\Delta A\right|}{A}=\pm \frac{0.55}{11.34}=\pm 5\%$$

The measurement uncertainty brought by material factors, radiation factors, and parts processing factors of *δ_A_* is included as well as the measurement uncertainty caused by waveform interpretation and time coordinate accuracy of $\delta_{\Delta t_{m}}$. The whole sensor is uncertain as follows:

(23) $$\delta_{I_{1}}\leq \pm {\left(0.1^{2}+4.2^{2}+5^{2}+12.6^{2}\right)}^{1/2}/100=\pm 14\%$$

## 4. Comparison of Experimental Results and Numerical Calculation under Thermodynamic Parameters of Electron Beam Machine

The numerical calculation of the electron beam blow-off impulse and the thermal shock wave peak stress was carried out by using the software of thermodynamic effects of pulsed beam irradiation [33,34,35]. By comparison, the calculation results were in good agreement with the experimental data. For the simulation experiment of electron beam irradiation, the electron beam is loaded according to the time course, with the electron beam diode current and voltage given by the experiment used as load conditions. With the voltage data corresponding to the electron beam energy, the energy deposition distribution of the electrons in the material can be obtained by the interpolation of the calculated energy deposition profile of the electron beam [36]. With the current data corresponding to the intensity of the electron beam, the energy flux at that time can be obtained by combining the transmission efficiency of the electron beam in the drift tube with the beam spot area reaching the target and the beam energy. The experimental state of electron beam can be basically simulated, which is to the greatest extent consistent with the experimental process [37].

**Figure 6:**
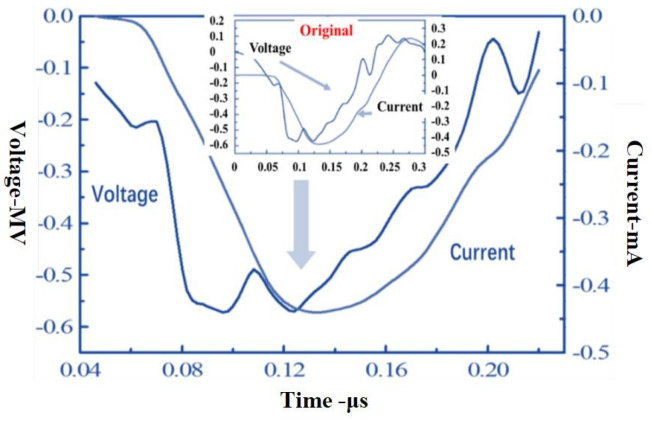
Original REB current and voltage waveforms from the experiment.

**Figure 7:**
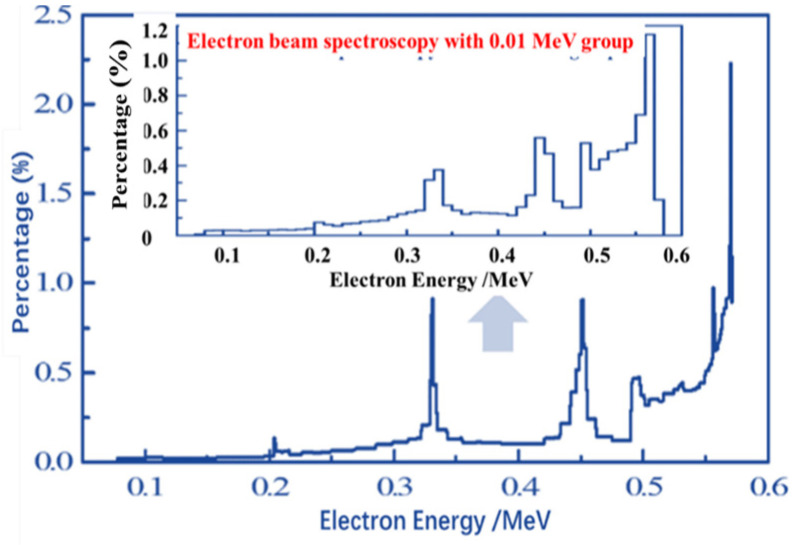
Electron beam energy spectrum from the experiment after 0.01 MeV grouping.

The small one is the original REB current and voltage waveforms from the experiment in Figure 6. The large one is extracted the valid pulse waveforms with negative current and voltage values in the same period, and it is applied in the calculation program. Figure 7 shows the electron beam energy spectrum—whose structure cannot be analyzed because of the small grouping energy and whose data not directly used in the numerical calculation are included in current and voltage values—from the experiment through the processing of the current and voltage data. Meanwhile, this spectrum is the one after 0.01 MeV grouping, from which the percentage of the electron beam in each energy range can be seen intuitively. The total beam intensity is measured by Rogowski coil, the current intensity is measured by Faraday array, and differential loop is used to measure REB diode voltage in experiment. The electron beam energy spectrum is obtained from current–voltage data processing. A special starting point is shown in the spectrum in Fig. 7, which is caused by a local increase in the energy density of the current voltage generated by the continuous discharge process of the capacitor combination. This phenomenon is the normal spectral input of REB and has no effect on the calculation result.

The chemical composition and physical properties of Al-alloy are shown in Table 1 [37] and Table 2 [38]. This Al-alloy material is the material used in the experiment, and these property parameters are also input into the RAMA program.

**Figure 8:**
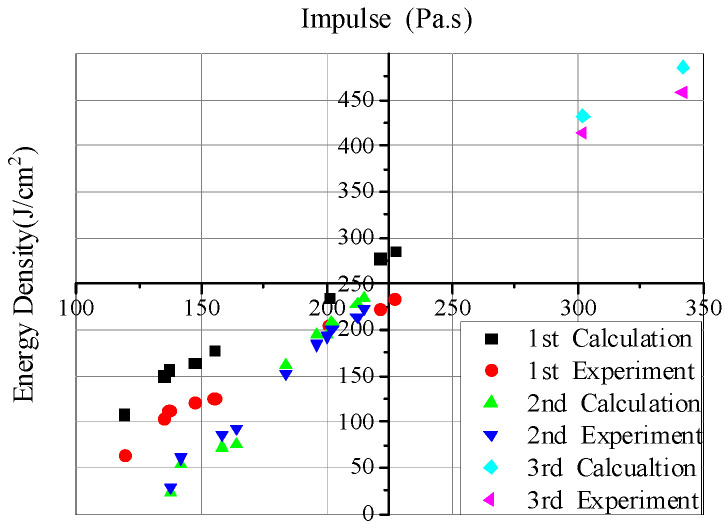
Comparison between calculated results and experimental data of blow-off impulse.

**Figure 9:**
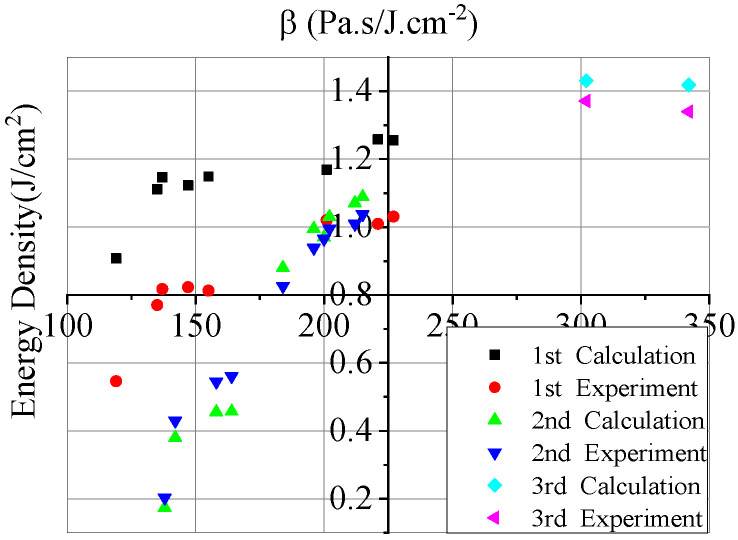
Comparison between calculated results and experimental data of impulse coupling coefficient.

According to the effective current and voltage data in the experiment, the transmission efficiency of the electron beam in the drift tube, and the beam spot area on the target surface, each experimental situation can be calculated by the finite difference method. Figure 8 shows the comparison between the calculated results and the experimental data, from which it can be seen that the former is mostly in good agreement with the latter. For the larger ones, they are still in reasonable agreement with consideration of the uncertain electron beam energy flux. In a word, the former, which is credible, is consistent with the latter in the range of experimental uncertainty, and the numerical calculation is carried out well by using the numerical calculation software. Figure 9 shows the comparison of the impulse coupling coefficients.

## 5. High Energy Flux Expansion Calculation

The limited effective data of thermodynamic experiment and the small range of energy flux fail to meet the requirements of equivalence research. Therefore, this paper expands the experimental results and its range by means of numerical calculation. The energy flux range for equivalence research needs to be extended to 500 J/cm^2^ from 100–250 J/cm^2^ for the experiment. First, the calculation approach for energy flux in the electron beam irradiation experiment needs to be analyzed.

(24) $$\delta_{I_{1}}\leq \pm {\left(0.1^{2}+4.2^{2}+5^{2}+12.6^{2}\right)}^{1/2}/100=\pm 14\%$$

where *U*(*i*) and *I*(*i*) represent the voltage and current data—including generally unchangeable system information of the electron beam accelerator—recorded in the experiment at each moment during the electron beam pulse irradiation; Δ*t* is the time interval of recording the current and voltage of the accelerator; and *k* is the transmission efficiency of the electron beam in the drift tube. The failure of online measurement triggers the values of these two changing in a small range. *R* is the radius of the electron beam spot on the target surface, with its size adjusted by changing the target position in the drift tube in the experiment. Moreover, different energy fluxes can be obtained by adjusting its size in the numerical calculation. This study gave out irradiation effects of the electron beam at high-energy flux by adjusting the beam spot radius and using numerical calculation for expansion. By taking the current and voltage as basic parameters and adjusting the beam spot radius, the blow-off impulse and thermal shockwave stress were experimentally calculated at the energy flux of 100–500 J/cm^2^, to complete the expansion of the experimental results of high-energy flux.

**Figure 10:**
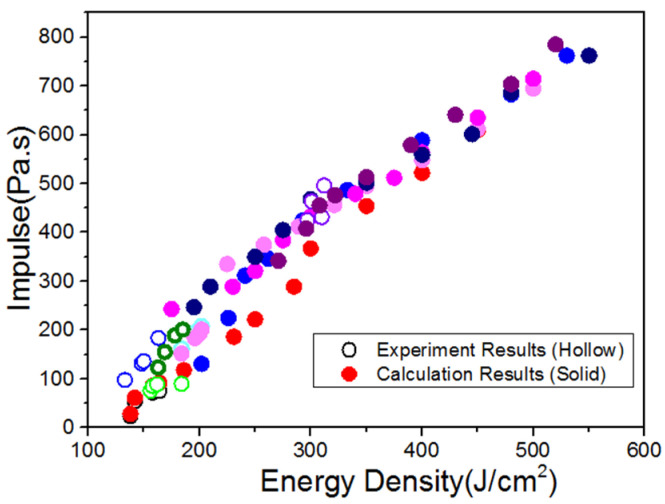
Calculation results of blow-off impulse by experimental expansion.

**Figure 11:**
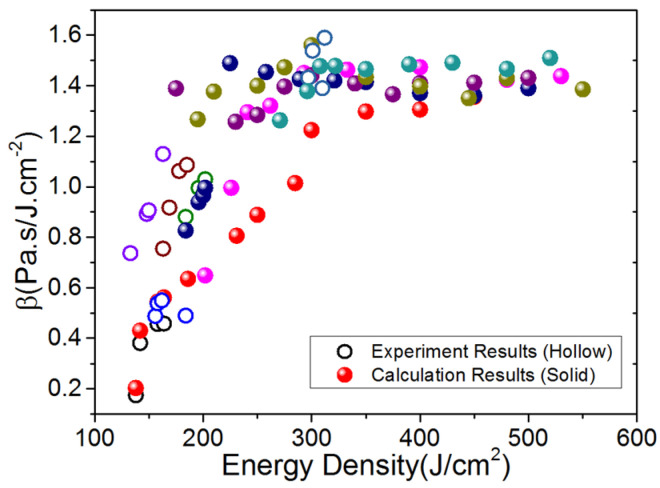
Calculation results of impulse coupling coefficient under different energy fluxes.

Figure 10 shows the calculation results of blow-off impulse by experimental expansion, under the electron beam irradiation blow-off impulse with energy flux ranging 100–500 J/cm^2^, with maintained system information of electron beam. The numerical calculation results are equivalent to the experimental ones to a certain extent. The blow-off impulse increases linearly with the change in energy flux. Figure 11 shows the calculation results and impulse coupling coefficients under different energy fluxes. The impulse coupling coefficient is consistent, according to the comparison between the experimental results and the calculated results in the same energy.

**Figure 12:**
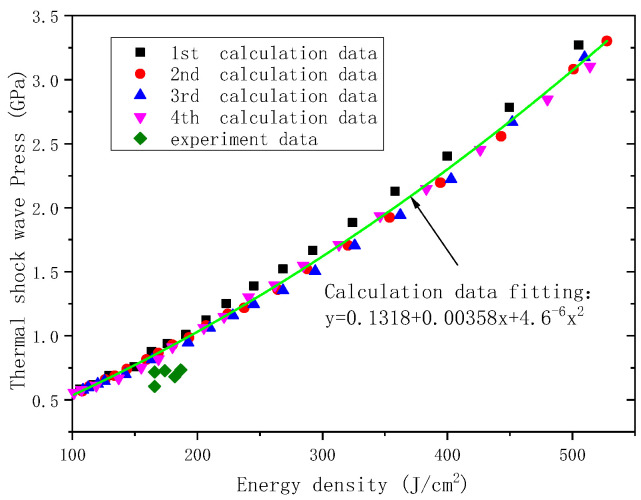
Calculation and experimental results of thermal shock wave under different energy fluxes.

Figure 12 shows the calculation results and experimental data of thermal shockwave stress under different energy fluxes. The former, albeit slightly larger, is in good agreement with the latter to a certain extent, with consideration of the uncertain energy flux and thermal shock wave stress. It shows the influence of uncertain electron beam system on the thermal shock wave stress, because of the linear increase in thermal shock stress with the change of the energy flux, and the small dispersion.

**Figure 13:**
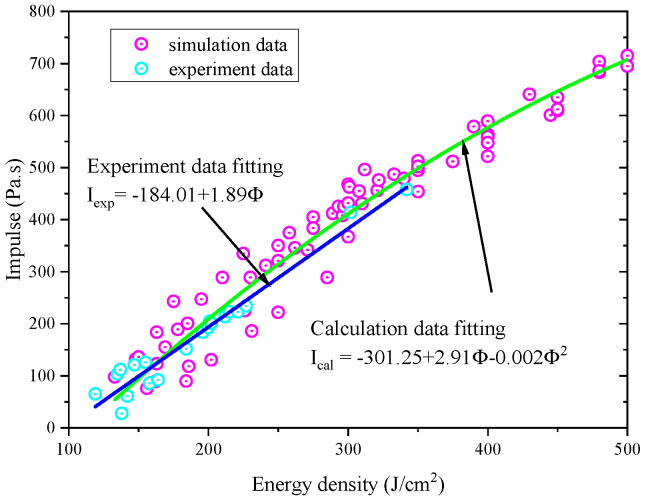
Calculation and experimental results and fitting curves of electron beam irradiation blow-off impulse under different energy fluxes.

To analyze the equivalence, the blow-off impulse results are calculated and compared with the experimental data, as shown in Figure 13. Meanwhile, the data are fitted and the formulas are given.

## 6. Numerical Calculation of X-ray Blow-Off Impulse

**Figure 14:**
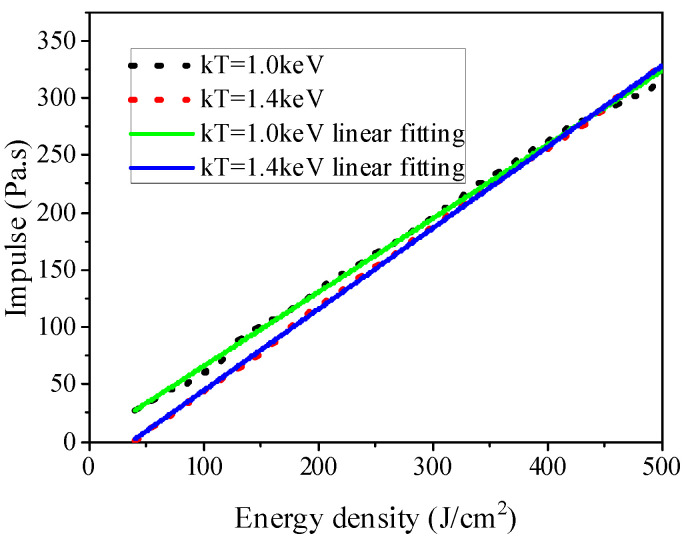
Calculation result and fitting curve of blow-off impulse of blackbody spectrum X-ray under different energy fluxes.

The same aluminum alloy materials were selected as the target materials in the experiment. The blackbody spectra were 1 keV and 1.4 keV, and the X-ray time spectrum used a rectangular pulse of 0.1 s in width. The variation of blow-off impulses with X-ray energy flux was calculated for X-ray spectrum, with its energy flux ranging 50–500 J/cm^2^. The calculation results of the blow-off impulse are shown in Figure 14, with the black and red lines representing the blow-off impulse of 1 keV and 1.4 keV blackbody spectrum X-rays, respectively. It can be seen from the graph that there is a different monotonous increase in blow-off impulse with the energy flux. The approximate fitting formulas are as follows:

(25) $$I_{kT=1.0}=0.65\Phi_{X}+2.0$$

(26) $$I_{kT=1.4}=0.7\Phi_{X}-25$$

## 7. Equivalence Relations between Electron Beam and Blackbody Spectrum X-ray Irradiation Blow-Off Impulse

According to the above calculation results of blow-off impulse, electron beam was adopted to simulate the blackbody spectrum X-ray irradiation blow-off impulse. After numerical analysis on the calculation results of both, it is found that there is a certain numerical correlation between the two blow-off impulses. Based on this, a simple numerical relation between the two blow-off impulses is as follows:

(27) $$I_{kT=1.0}=0.4I_{electron}+35\;(\mathrm{Pa}\cdot s)$$

(28) $$I_{kT=1.4}=0.43I_{electron}+10\;(\mathrm{Pa}\cdot s)$$

**Figure 15:**
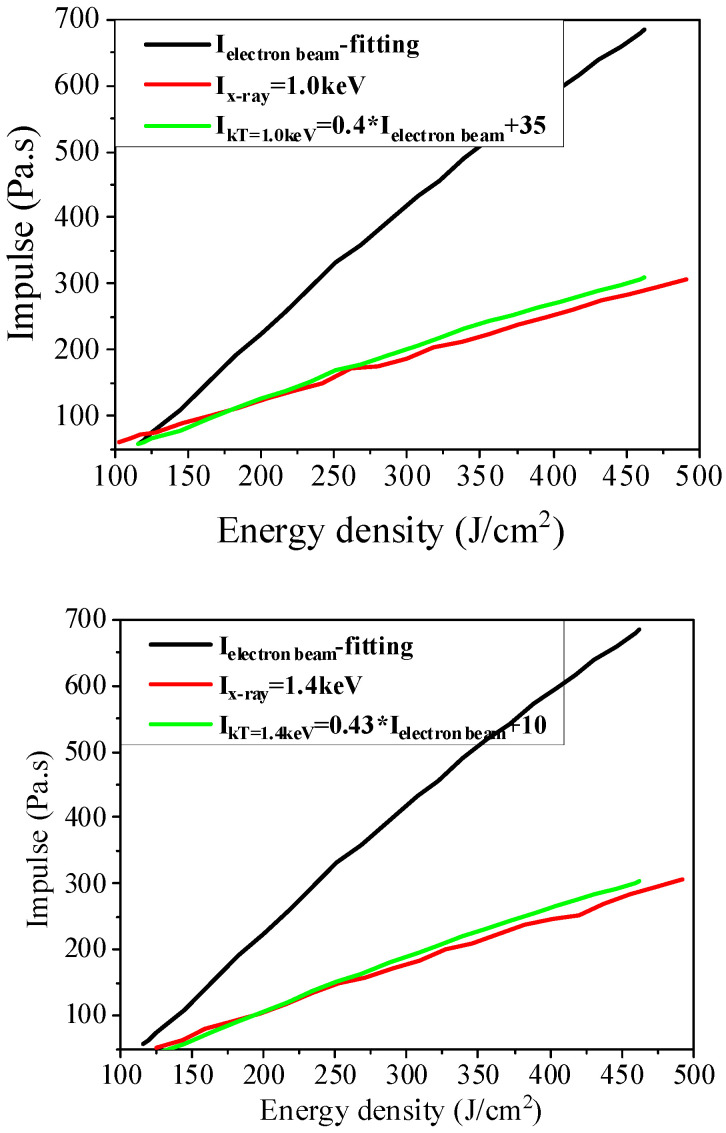
Comparison between the results of energy–flux relation conversion and blackbody spectrum X-ray irradiation blow-off impulse.

This formula provides the numerical relations between the irradiation blow-off impulse of the electron beam and the blackbody spectrum X-ray under the same energy flux, which is called equivalent energy flux relations. Figure 15 shows the comparison between the numerical calculation results and the blackbody spectrum X-ray’s irradiation blow-off impulse converted from that of the electron beam under identical energy flux relations. It can be seen intuitively that they are in good agreement.

Formula (29) exhibits the numerical relation between electron beam and blackbody spectrum X-ray irradiation blow-off impulse at the same energy flux. However, it is not intuitive and convenient to directly apply it to equivalence and provide equivalence principles. Therefore, a further analysis of the relation between the data for the two blow-off impulses obtained the energy flux relation at the same blow-off impulse by the irradiation of electron beam and blackbody spectrum X-ray.

(29) $$\Phi_{KT=1.0}=2.5\Phi_{electron}-130\;(J/{\mathrm{cm}}^{2})$$

(30) $$\Phi_{kT=1.4}=2.3\Phi_{electron}-80\;(J/{\mathrm{cm}}^{2})$$

**Figure 16:**
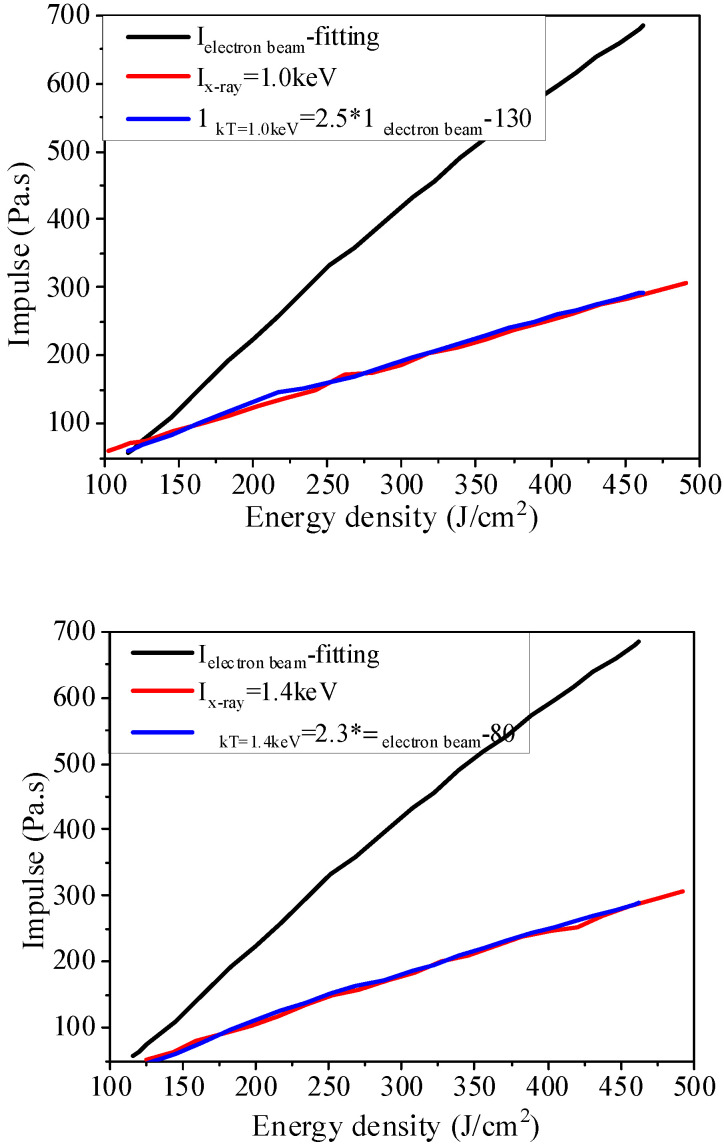
Comparison between equivalent impulse-based conversion results and blackbody spectrum X-ray irradiation blow-off impulse.

The above formula reveals the numerical relations of energy flux at the same blow-off impulse by the irradiation of electron beam and blackbody spectrum X-ray, which is called equivalent impulse relations. From the comparison between the numerical results and the blackbody spectrum X-ray’s blow-off impulse converted from that of electron beam as per the above equivalent impulse relations, as shown in Figure 16, it can be found that they are in good agreement.

**Figure 17:**
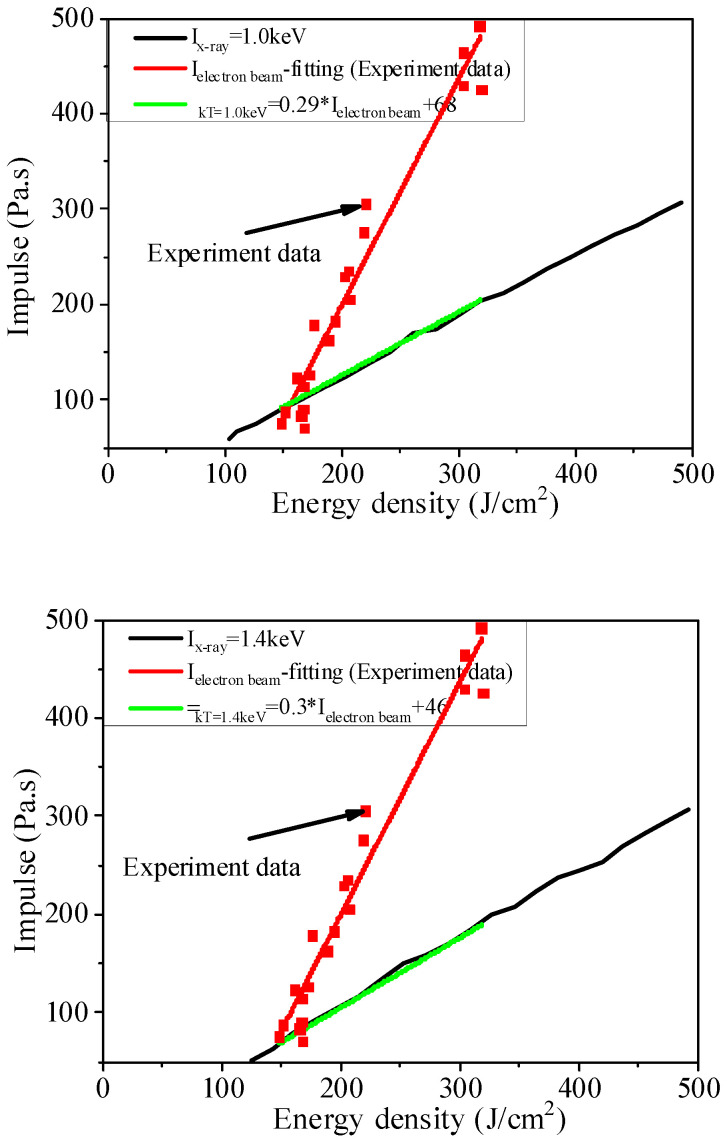
Comparison between the fitting curves of experimental blow-off impulse after conversion via the equivalent energy flux relations and the blackbody spectrum X-ray irradiation blow-off impulse.

Figure 17 shows the equivalent energy flux relations for measurement results of the experimental blow-off impulse. The results from comparison show that the simulation results of blackbody spectrum X-ray irradiation blow-off impulse converted from the experimental results of blow-off impulse as per the above flux relations are in great agreement with the numerical results. The experimental results of blackbody spectrum X-ray irradiation blow-off impulse at the same energy flux can be predicted according to the blow-off impulse by the electron beam experiment and the above flux relations. The experimental results of blackbody spectrum X-ray irradiation blow-off impulse could be indirectly obtained by the electron beam simulation experiment, in case of the lack of blackbody spectrum X-ray irradiation sources.

(31) $$I_{kT=1.0}=0.29I_{{}_{electron(experimen)}}+68\;(\mathrm{Pa}\cdot s)$$

(32) $$I_{kT=1.4}=0.3I_{{}_{electron(experiment)}}+46\;(\mathrm{Pa}\cdot s)$$

**Figure 18:**
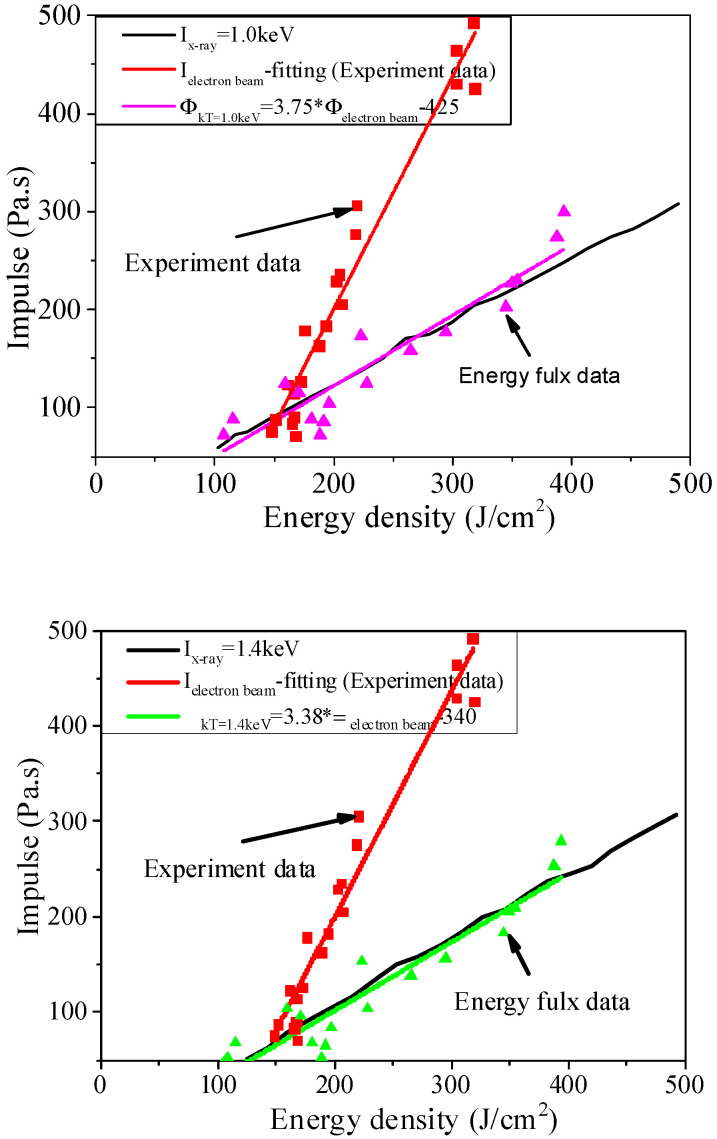
Comparison between the fitting curves of experimental blow-off impulse after conversion via equivalent impulse relations and the blackbody X-ray irradiation blow-off impulse.

Figure 18 shows an equivalent impulse relation, which is a direct equivalent one. The results obtained by numerical calculation are in good agreement with those obtained by X-ray irradiation. The electron beam experiment can be designed using the equivalent impulse relations. The blackbody spectrum X-ray irradiation blow-off impulse value by direct measurement under the concerned flux is equivalent to the experimental result of blackbody spectrum X-ray irradiation blow-off impulse directly obtained by the electron beam experiment without sufficient blackbody spectrum X-ray irradiation sources.

(33) $$\Phi_{kT=1.0}=3.75\Phi_{electron(\exp eriment)}-425\;(J/{\mathrm{cm}}^{2})$$

(34) $$\Phi_{kT=1.4}=3.38\Phi_{electron(\exp eriment)}-340\;(J/{\mathrm{cm}}^{2})$$

## 8. Conclusions

This paper studied the equivalence problems in thermodynamics effects between the multienergy composite spectrum electron beam and blackbody spectrum X-ray, which could provide a reference for rapid determination of experimental parameters in simulation experiments.

(1) The electronic beam experimental platform and impulse measurement system were introduced to analyze the set input parameters by numerical calculation on the basis of experimental conditions. By comparison of the impulse coupling coefficient, it was shown that the credible calculation results were consistent with the experimental data in the range of experimental uncertainty. The numerical simulation was carried out well using numerical calculation software.

(2) Experiment results and energy fluence range were expanded by numerical calculation to meet the requirements of equivalence study, and the data for electron beam irradiation blow-off impulse with energy fluence of 100–500 J/cm^2^ were thus obtained. This result, meanwhile, maintained the input information of the electron beam system. From impulse calculation results, the blow-off impulse presented a linear increase with energy fluence.

(3) The RAM software with a self-developed program was employed to achieve the X-ray irradiation blow-off impulse and the approximate fitting formula under blackbody spectra of 1 keV and 1.4 keV. For input conditions, aluminum alloy—the same as the target material for the experimental measurement—was selected, while the rectangular pulse of 0.1 μs in width was adopted for the X-ray time spectrum, thus obtaining the energy fluence of 50–500 J/cm^2^.

(4) By comparison between the experiment results of the electron beam and the blackbody spectrum X-ray numerical simulation, the equivalent flux and impulse relations were obtained.

In a word, based on the actual results of the electron beam simulation experiment, the results were also expanded by numerical calculation. The numerical calculation of the blackbody spectrum X-ray irradiation blow-off impulse gave out the variation curve of the X-ray blow-off impulse with the energy flux. Based on this, the equivalent flux and impulse relations were obtained by analyzing the numerical and experimental calculation results of the electron beam blow-off impulse and the calculation results of the X-ray blow-off impulse. Through such relations, the experimental results of blackbody spectrum X-ray irradiation blow-off impulse could be obtained directly or indirectly by the electron beam simulation experiment. This is of certain practical significance.

## Figures and Tables

**Table 1:** Chemical composition of Al-alloy (wt %).

Fe	Si	Cu	Mn	Mg	Zn	Ti	Ni	Others	Al
0.14	0.06	4.5	0.83	1.6	0.09	0.04	0.08	<0.05	Surplus

**Table 2:** Physical properties of Al-alloy.

Specific Heat Capacity /(J·kg^−1^·K^−1^)	Coefficient of Thermal Expansion /(10^−6^K^−1^)	Thermal Conductivity /(W·m^−1^·K^−1^)	Density /(kg·m^−3^)	Young’s Modulus/GPa	Poisson’s Ratio
900	23	143	2780	70	0.33

## Data Availability

All data included in this study are available upon request by contact with the corresponding author.

## References

[1] Office of the Deputy Assistant Secretary of Defense for Nuclear Matters (ODASD(NM)) Nuclear Matters Handbook 2020. www.acq.osd.mil/ncbdp/nm.

[2] Qiao D.J. (2012). Thermodynamic Effect and Reinforcing Technology under Pulse X-ray Radiation.

[3] Glasstone S.A. (1977). The Effects of Nuclear Weapons.

[4] Langley R.W. (1974). Analytical Relationships for Estimating the Effects of X-rays on Materials.

[5] Lawrence R.J. (1992). The equivalence of simple models for radiation-induced impulse. Shock Compression of Condensed Matter–1991.

[6] Benham R.A. (1997). Reentry vehicle cold X-ray induced impulse technology development: Materials blow-off characteristics. Modeling, and Experimental Facilities.

[7] Rivera W.G., Benham R.A. (1999). Explosive Technique for Impulse Loading of Space Structures.

[8] Briggs E.A., Veigele W.J. (1971). X-ray cross Section Compilation from 0.1 keV to 1 MeV.

[9] Chena J.K., Berauna J.E., Grimesa L.E. (2002). Modeling of femtosecond laser induced non-equilibrium deformation in metal films. Int. J. Solids Struct..

[10] Karakas A., Tunc M., Camdali C.U. (2010). Thermal analysis of thin multi-layer metal films during femtosecond laser heating. Heat Mass Transf..

[11] Hettche L.R., Tucker T.R., Schrempf J.T. (1976). Mechanical response and thermal coupling of metallic targets to high-intensity 1.06-μm laser radiation. J. Appl. Phys..

[12] Doron K., Eli W. (2010). Hard X-ray emission from accretion shocks around galaxy clusters. J. Cosmol. Astropart. Phys..

[13] Zhu J., Yu H.J., Chen N. (2011). Hydrodynamic response of converter target impacted by high current relativistic electron beam. Nucl. Instrum. Methods Phys. Res. Sect. B.

[14] Ramirez J.J., Rrestwich K.R., Johnson D.L., Corley J.P., Denison G.J., Huddle C.W., Pate R.C., Weber G.J., Burgess E.L., Hamil R.A. Hermes III: A TW, short pulse, gamma ray simulato. Proceedings of the 7th Pulsed Power Conference.

[15] Kovalcud B.M., Mesyats G.A. Generation of High-Power Pulsed Microwaves. Proceedings of the Eight International Conference on High-Power Particle Beams.

[16] Hu Z.Y., Zhang Z.H., Cheng X.W. (2020). A review of multi-physical fields induced phenomena and effects in spark plasma sintering: Fundamentals and applications. Mater. Des..

[17] Guilherme P.S., Rafael H.M., Sheila M.C. (2019). Weldability of a zirconium alloy comparing resistance and pulsed laser methods. Nucl. Mater. Energy.

[18] Li M.Y., Sommerer M., Werner E. (2015). Experimental and computational study of damage behavior of tungsten under high energy electron beam irradiation. Eng. Fract. Mech..

[19] Office of the Under Secretary of Defense (2005). Report of the Defense Science Board Task Force on Nuclear Weapon Effects Test, Evaluation, and Simulation. www.bits.de/NRANEU/docs/NW-Effects05.pdf.

[20] Venkata K.A., Truman C.E., Smith D.J. (2016). Characterizing electron beam welded dissimilar metal joints to study residual stress relaxation from specimen extraction. Int. J. Press. Vessel. Pip..

[21] Leggieri A., Passi D., Paolo F.D. (2015). Design of a sub-millimetric electron gun with analysis of thermomechanical effects on beam dynamics. Vacuum.

[22] Milov I., Lipp V., Ilnitsky D. (2020). Similarity in ruthenium damage induced by photons with different energies: From visible light to hard X-rays. Appl. Surf. Sci..

[23] Fitting H.J. (2007). Electron beam excitation in thin layered samples. J. Electron Spectrosc. Relat. Phenom..

[24] Hu L., Lei Y., Zhu J. (2013). Simulation on distributed target material impacted by high intensity current multi-pulse electron beam. High Power Laser Part. Beams.

[25] Lukyanov A.A. (2008). Constitutive behavior of anisotropic material under shock loading. Int. J. Plast..

[26] Zhou N., Qiao D.J. (2002). Materials Dynamics under Pulse Beam Radiation.

[27] Rudie N.J. (1976). Principles and Techniques of Radiation Hardening.

[28] Tang W.H., Zhang R.Q. (1997). Numerical research on the destructibility of pulsed electron beam to materials. High Power Laser Part. Beams.

[29] Chavez M.A., Covert T.T. (2013). Synthesis Microstructure and Explosive Properties of Spray-Deposited Silver Acetylide-Silver Nitrate Composite Light Initiated High Explosives.

[30] Chavez M.A. (2012). Implications of Explosively Accelerating Thin Flyer Plates in the Transient Regimes of Explosive Systems. Ph.D. Thesis.

[31] Chen Z.L., Peng S.M., Meng D. (2014). Monte Carlo calculation of energy deposition in ionization chambers for tritium measurement. Nucl. Instrum. Methods Phys. Res. A.

[32] Wu D.F., Xu X.F., Zhang L. (2016). A hybrid Monte Carlo model for the energy response functions of X-ray photon counting detector. Nucl. Instrum. Methods Phys. Res. A.

[33] Sun J.F., Hu Y., Sun L. (2015). An average method for intense pulsed electron beam incident angles. High Power Laser Part. Beams.

[34] Yang H.L., Qiu A.C., Zhang J.S. (2002). Simulation calculation for the energy deposition profile and the transmission fraction of intense pulsed electron beam at various incident angles. High Power Laser Part. Beams.

[35] Liu G.Z. (2003). Numerical simulation on the energy spectrum of the electron beam generated by low-impedance diode and the influence of external magnetic field on diode impedance. High Power Laser Part. Beams.

[36] Hu Y., Yang H.L., Sun J.F. (2015). A method of measuring the incidence angle of intense electron beam. Acta Phys..

[37] Xiao L.J., Song W.D., Wang C. (2017). Mechanical properties of open-cell rhombic dodecahedron titanium alloy lattice structure manufactured using electron beam melting under dynamic loading. Int. J. Impact Eng..

[38] Wen B.C. (2014). Machine Design Handbook.

